# Safe Pseudovirus-based Assay for Neutralization Antibodies against Influenza A(H7N9) Virus

**DOI:** 10.3201/eid1910.130728

**Published:** 2013-10

**Authors:** Chao Qiu, Yang Huang, Anli Zhang, Di Tian, Yanmin Wan, Xiaoling Zhang, Wanju Zhang, Zhiyong Zhang, Zhenghong Yuan, Yunwen Hu, Xiaoyan Zhang, Jianqing Xu

**Affiliations:** Shanghai Public Health Clinical Center, Shanghai, China (C. Qiu, A. Zhang, D. Tian, Y. Wan, Xiaoling Zhang, W. Zhang, Z. Zhang, Z. Yuan, Y. Hu, Xiaoyan Zhang, J. Xu);; Fudan University, Shanghai (C. Qiu, Y. Huang, A. Zhang, Z. Yuan, Xiaoyan Zhang, J. Xu);; Chinese Center for Disease Control and Prevention, Beijing, China (Xiaoyan Zhang, J. Xu)

**Keywords:** Influenza, subtype H7N9, serology, neutralization tests, pseudovirus, viruses

## Abstract

Serologic studies are urgently needed to assist in understanding an outbreak of influenza A(H7N9) virus. However, a biosafety level 3 laboratory is required for conventional serologic assays with live lethal virus. We describe a safe pseudovirus–based neutralization assay with preliminary assessment using subtype H7N9–infected samples and controls.

A novel reassortant avian influenza A(H7N9) virus has emerged in eastern China and caused fatal infections in humans (*1**–**3*). The real-time reverse-transcription PCR (RT-PCR) is used as a diagnostic method for detection of subtype H7N9 in infected patients or birds within the window of time when virus shedding is expected. Because of the pathogen-specific immune memory response in persons with asymptomatic cases or patients who have cleared infection, serologic assays are invaluable tools for estimating the incidence and prevalence in the population affected by the outbreak. However, those studies were confined to conventional hemagglutination-inhibition (HI) or microneutralization (MN) assays that are limited to propagation of the live lethal subtype H7N9 influenza virus in biosafety level 3 (BSL-3) laboratories. We describe a rapid and safe pseudovirus-based assay for detecting subtype H7N9 neutralizing antibodies. This assay can be performed in most laboratories equipped with BSL-2 facilities.

## The Study

Using a previously reported method (*4*), we assembled the pseudovirus with membrane proteins of hemagglutinin (HA) and neuraminidase (NA) from influenza A/Shanghai/4664T/2013(H7N9) and capsid protein from HIV. The genomic RNA of the pseudovirus also carries a luciferase reporter gene; thus its infectivity can be quantified by luciferase activity in virus-infected cells (*5**–**7*). To produce the pseudovirus, we carried out the following procedures: 4.5 × 10^6^ 293T cells cultured in a 10-cm dish were co-transfected with 5 μg of lentivirus vector pNL4-3-Luc R-E-, 2.5 μg of pVKD-HA, and 2.5 μg of pVKD-NA by using lipofectamine 2000 from Invitrogen (cat no. 11668; Carlsbad, CA, USA). The coding sequences of HA (GenBank accession no. KC853228) and NA (GenBank accession no. KC853231) were amplified from A/Shanghai/4664T/2013(H7N9) by using RT-PCR. (These constructs of pVKD-HA and pVKD-NA can be provided on request from the authors.) After overnight incubation, cells were washed once with phosphate-buffered saline and cultured in 5 mL complete Dulbecco minimum essential medium. The pseudovirus containing supernatant was harvested at 48 hours and stored at −80°C in aliquots until used in the neutralization assay. MDCK cells were infected with serially diluted pseudovirus stock, and the infectivity reflected by the relative luciferase activity (RLA) was determined as the median (50%) tissue culture infective dose, according to the method of Reed and Muench.

We then implemented the pseudovirus assay to measure neutralizing antibodies in clinical samples with known HI titers for subtype H7N9. Fourteen convalescent-phase serum samples with real-time RT-PCR–confirmed infection with 2013 subtype H7N9 were from patients 56–81 years of age from whom samples were collected 12–32 days after onset of symptoms and who were hospitalized in Shanghai Public Health Clinical Center; control samples were 50 stored serum samples collected in 2009 before the emergence of 2013 subtype H7N9. The neutralizing activities in patients’ serum samples were also validated by the MN assay against live virus. In a 96-well plate, 2-fold serially diluted serum samples beginning at 1:10 were incubated with 200 median (50%) tissue culture infective doses of pseudovirus at the final volume of 100 μL at 37°C for 1 hour; then the mixture was added to the culture of MDCK cells. After incubation overnight, the cells were washed with 200 μL of phosphate-buffered saline and cultured in complete Dulbecco minimum essential medium for 48 hours in the original 96-well plate. RLA was measured by BrightGlo luciferase substrate (Promega, Madison, WI, USA; cat. no. E2610). Inhibition percentage was calculated as the following: (RLA in virus challenge controls – RLA in test well for each serum at specific dilution)/RLA in virus challenge controls. The 50% inhibitory concentration (IC_50_) titer was determined by the reciprocal of the last dilution that resulted in >50% reduction of luciferase activity. As expected, the IC_50_ titer quantified by the pseudovirus-based assay significantly correlated with HI titer measured by the inhibition of 1% guinea pig erythrocytes hemagglutination when incubated for 1 hour with reacted mixture of diluted serum samples and live virus (Figure; Spearman r = 0.88, p<0.0001, n = 64). 

**Figure:**
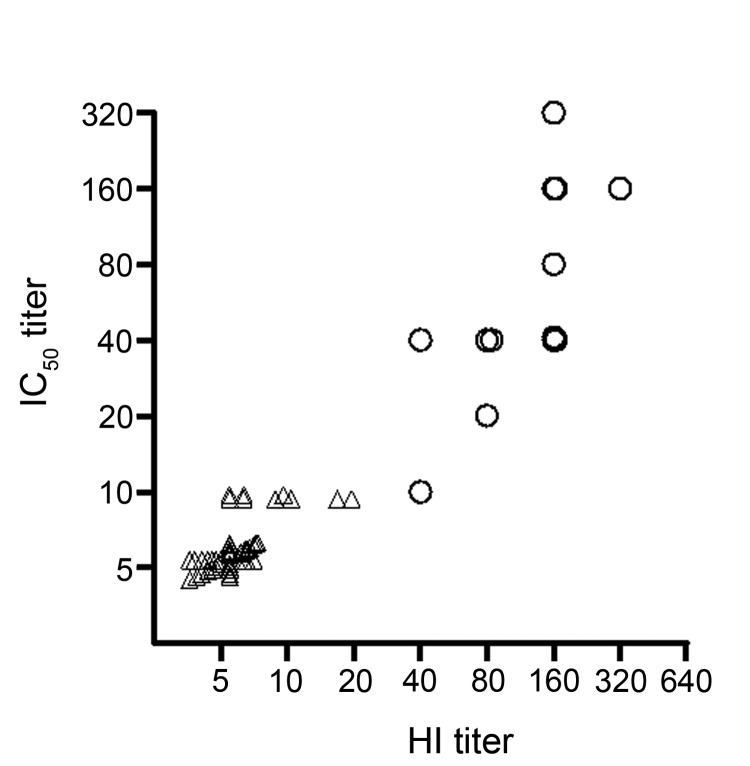
Correlation between conventional hemagluttination (HI) titer and 50% inhibitory concentration (IC_50_) titer of pseudovirus-based assay for diagnosing influenza A(H7N9) virus infection. Correlation between the IC_50_ titer of the pseudovirus-based neutralization assay and the titer of conventional HI assay, tested with 14 serum samples collected >10 days after symptom onset from patients with real-time RT-PCR–confirmed 2013 influenza A(H7N9) infection (○) and 50 control samples (△, Spearman r = 0.88, p<0.0001, n = 64). To display the information from all the samples, overlapped markers were shifted on the x- and/or y-axis with small incremental units.

To determine the criteria delineating seropositive samples from seronegative samples for the pseudovirus-based assay, we tested the agreement between the HI assay and pseudovirus-based assay with the thresholds of 20 and 40, respectively. The strongest concordance between these assays was achieved by using 40 for the pseudovirus-based assay as the cutoff titer (Table; κ = 0.904; McNemar test, p = 0.5 for 1:40 and κ = 0.788; McNemar test, p = 0.375 for 1:20). With 40 as the cutoff, 12 (86%) of 14 HI assay samples positive for subtype H7N9 had an IC_50_ titer >40, whereas none of the control samples had an IC_50_ titer >40 (Table). Thus, in our preliminary assessment using this limited number of samples, the sensitivity of this assay was 85.7 (95% CI 0.572–0.982), and the specificity was 1.000 (95% CI 0.929–0.978). In addition, the serum samples used as negative controls contained antibodies against contemporary circulating seasonal influenza viruses, including H1 and H3 subtypes, which also supported the strain specificity of this subtype H7N9 neutralization assay (data not shown).

**Table:** Comparison of HI and pseudovirus-based neutralization assay results for influenza A(H7N9) virus*

HI titer†	Pseudovirus IC_50_ titer			
>40	<40‡	>20§	<20§
+	12	2	13	1
–	0	50	4	46

*Values are no. samples. HI, hemagglutination inhibition; IC50, 50% inhibitory concentration. †+ HI titer is >40; – HI titer is <40. ‡κ = 0.904; McNemar test, p = 0.5. §κ = 0.788; McNemar test, p = 0.375.

## Conclusions

Here we provide an alternative approach for quantifying antibody responses to the new avian influenza A(H7N9) virus. Conventional HI or MN assays require propagating the live subtype H7N9 virus, which is known as a lethal virus. The pseudovirus can infect in a single round, which is much safer than handling the highly virulent subtype H7N9 virus. All of these processes for the pseudovirus-based neutralization assay can be performed at routine BSL-2 settings, and most field laboratories equipped with mammalian cell culture facilities meet this biosafety requirement. Moreover, propagating avian influenza virus in embryonic eggs to high titer is labor intensive and time consuming and requires empirical judgment to interpret the results of HI and MN assays. By contrast, large amounts of pseudoviruses take only 2–3 days to produce, and using RLA as the readout of pseudovirus-based neutralization assay provides an objective means of interpreting the endpoints. 

Therefore, this pseudovirus-based neutralization assay could be used as an alternative for safely conducting serologic studies in a rapid response in assessing the threat posed by subtype H7N9 virus. Of note, the pseudovirus-based assay was less sensitive than HI assay when tested by our small number of samples, indicating that some false-negative results would be observed and thus that some samples that would test positive by HI might be missed. The differences between the 2 assays could be explained by the possibility that HI and pseudovirus-based assays evaluate different components of the antibody response. HI only measures the proportion of antibodies directed to the receptor-binding site of hemagglutinin, whereas the neutralization assay detects a broader range of neutralizing antibodies. The results of these 2 assays were in agreement for most samples, indicating that 2 different components of antibodies are likely to be developed in parallel in most persons. Therefore, this assay will provide a new contribution to the understanding of how the immune system responds to infection with influenza viruses.
